# Supplementary material for: Impact of a Rice-Centered Diet on the Quality of Sleep in Association with Reduced Oxidative Stress: A Randomized, Open, Parallel-Group Clinical Trial
Source: Nutrients. 2020 Sep 24;12(10):2926. doi: 10.3390/nu12102926 (PMC7650672; doi:10.3390/nu12102926)
Supplement: Supplementary file 1 [file nutrients-12-02926-s001.pdf]

Table S1. Changes in food intake during intervention

Food item in BDHQ	Group	Duration of intervention (g/day/1000kcal) 0 weeks	4 weeks	8 weeks	p–values* Group–by–time interaction
Low fat milk	No-rice-diet	24.3 (9.1 – 39.5)	34.5 (10.2 – 58.8)	19.8 (5.7 – 34)	0.71
	Rice-diet	27.0 (6.8 – 47.1)	27.8 (6.1 – 49.5)	22.2 (3.4 – 40.9)	
Normal/high fat milk	No-rice-diet	64.4 (31.3 – 97.5)	59.6 (36.8 – 82.4)	71.9 (49.9 – 93.9)	0.92
	Rice-diet	33.3 (16.7 – 49.8)	29.6 (12.3 – 46.9)	37.6 (19.5 – 55.7)	
Chicken	No-rice-diet	27.1 (20.2 – 34.0)	23.8 (18.3 – 29.3)	30.0 (23.1 – 36.9)	0.86
	Rice-diet	19.8 (14.6 – 25.0)	17.3 (13.3 – 21.3)	21.6 (15.4 – 27.7)	
Pork/beef	No-rice-diet	30.6 (23 – 38.2)	27.4 (21.3 – 33.5)	29.8 (23.1 – 36.5)	0.28
	Rice-diet	36.9 (26.2 – 47.5)	24.6 (19.0 – 30.3)	25.8 (21.0 – 30.6)	
Ham/Sausage	No-rice-diet	12.8 (8.0 – 17.5)	9.3 (6.9 – 11.6)	9.8 (7.3 – 12.4)	0.69
	Rice-diet	9.9 (6.7 – 13.2)	8.5 (5.9 – 11.2)	9.2 (6.2 – 12.1)	
Liver	No-rice-diet	0.7 (0.2 – 1.2)	0.6 (0.2 – 1.1)	0.8 (0.3 – 1.4)	0.59
	Rice-diet	0.7 (0.1 – 1.3)	0.4 (0.0 – 0.7)	0.4 (−0.1 – 0.9)	
Squid/Octpus/ Shrimp/Shellfish	No-rice-diet	15.8 (11.9 – 19.8)	19.0 (11.9 – 26.1)	17.0 (11.1 – 23.0)	0.51
	Rice-diet	15.8 (11.5 – 20.0)	13.5 (9.5 – 17.5)	16.8 (10.6 – 23.1)	
Fish (with edible bones)	No-rice-diet	3.9 (2.4 – 5.4)	3.7 (1.8 – 5.6)	6.9 (3.3 – 10.6)	**0.023**
	Rice-diet	7.0 (3.3 – 10.8)	2.9 (1.8 – 4.1)	3.9 (2.4 – 5.3)	
Tuna(canned)	No-rice-diet	2.8 (1.4 – 4.2)	2.2 (1.4 – 3.0)	3.0 (1.7 – 4.3)	0.86
	Rice-diet	3.3 (1.6 – 5.1)	2.9 (0.9 – 5.0)	3.4 (1.4 – 5.4)	
Dried fish	No-rice-diet	27.6 (21.3 – 34.0)	22.4 (16.1 – 28.7)	22.8 (16.7 – 28.9)	0.33
	Rice-diet	20.5 (13.1 – 27.8)	22.0 (14.2 – 29.7)	22.4 (16.3 – 28.5)	
High–fat fish	No-rice-diet	16.9 (11.5 – 22.3)	18.2 (13.6 – 22.8)	15.0 (11.4 – 18.7)	0.39
	Rice-diet	19.5 (12.7 – 26.2)	16.4 (11.7 – 21.1)	14.3 (10.2 – 18.3)	
Low–fat fish	No-rice-diet	23.5 (16.3 – 30.7)	16.1 (10.3 – 21.9)	15.6 (9.5 – 21.7)	**0.015**
	Rice-diet	17.4 (13.2 – 21.6)	20.4 (14.7 – 26.1)	19.4 (13.3 – 25.6)	
Egg	No-rice-diet	42.2 (30.7 – 53.6)	42.5 (33.6 – 51.3)	41.8 (32.4 – 51.2)	0.52

	Rice-diet	32.3 (24.0 – 40.6)	37.4 (28.9 – 45.8)	39.9 (29.7 – 50.1)	
Tofu	No-rice-diet	44.3 (35.9 – 52.7)	41.3 (30.7 – 51.9)	38.9 (30.6 – 47.2)	0.68
	Rice-diet	31.0 (24.7 – 37.3)	30.3 (21.1 – 39.5)	30.3 (22.3 – 38.3)	
Fermented soybean	No-rice-diet	14.4 (9.4 – 19.4)	11.0 (6.8 – 15.1)	12.1 (7.6 – 16.5)	0.058
	Rice-diet	18.1 (12.9 – 23.3)	20.7 (15 – 26.4)	18.4 (13.2 – 23.6)	
Potato	No-rice-diet	55.8 (34.3 – 77.3)	44.4 (33.4 – 55.4)	53.0 (40.0 – 65.9)	0.055
	Rice-diet	41.3 (31.7 – 50.9)	58.2 (35.1 – 81.2)	45.9 (32.2 – 59.6)	
Pickled vegetables (greens, carrots, etc.)	No-rice-diet	11.0 (6.3 – 15.7)	7.0 (4.4 – 9.6)	6.5 (4.3 – 8.7)	0.067
	Rice-diet	4.7 (2.8 – 6.6)	5.8 (3.7 – 7.9)	6.8 (4.9 – 8.6)	
Pickled vegetables (light–colored vegetables)	No-rice-diet	8.6 (4.7 – 12.5)	6.4 (3.9 – 8.8)	7.7 (5.0 – 10.5)	0.34
	Rice-diet	9.4 (5.3 – 13.4)	11.2 (6.6 – 15.9)	11.1 (6.0 – 16.2)	
Fresh vegetables	No-rice-diet	32.9 (24.1 – 41.7)	32.4 (24.6 – 40.2)	29.6 (22.6 – 36.6)	0.87
	Rice-diet	25.2 (18.1 – 32.2)	27.5 (18.7 – 36.2)	23.2 (19.1 – 27.4)	
Greens	No-rice-diet	36.7 (23.9 – 49.5)	25.4 (18.7 – 32.0)	23.9 (16.7 – 31.0)	0.32
	Rice-diet	20.5 (15.4 – 25.6)	17.1 (12.7 – 21.5)	19.0 (12.4 – 25.6)	
Cabbage	No-rice-diet	47.6 (36.6 – 58.7)	35.6 (25.2 – 46.0)	36.2 (27.4 – 45.1)	0.44
	Rice-diet	36.5 (25.2 – 47.7)	30.1 (23.4 – 36.7)	35.5 (28.3 – 42.7)	
Carrots/Pumpkins	No-rice-diet	23.5 (15.3 – 31.6)	18.4 (12.5 – 24.2)	15.4 (11.9 – 18.9)	0.46
	Rice-diet	15.7 (11.8 – 19.5)	14.3 (9.9 – 18.6)	13.9 (9.8 – 18.1)	
Radish/Turnip	No-rice-diet	30.0 (17.6 – 42.4)	23.7 (14.8 – 32.7)	21.9 (16.0 – 27.7)	0.16
	Rice-diet	15.4 (11.2 – 19.6)	21.0 (14.6 – 27.5)	18.9 (13.4 – 24.4)	
Other root vegetables	No-rice-diet	37.9 (29.2 – 46.7)	34.6 (25.7 – 43.4)	30.5 (23.9 – 37.2)	0.91
	Rice-diet	37.2 (29.3 – 45.1)	35.7 (24.6 – 46.9)	29.1 (21.8 – 36.4)	
Tomato	No-rice-diet	36.5 (22.5 – 50.4)	22.6 (12.1 – 33)	18.0 (8.7 – 27.4)	0.11
	Rice-diet	23.4 (16.4 – 30.3)	14.6 (9.0 – 20.3)	18.2 (12.2 – 24.2)	
Mushrooms	No-rice-diet	9.4 (6.7 – 12.1)	8.0 (5.9 – 10.0)	6.9 (4.8 – 9.1)	0.10
	Rice-diet	8.7 (6.5 – 10.9)	7.3 (5.2 – 9.4)	9.0 (6.4 – 11.5)	
Seaweeds	No-rice-diet	10.6 (5.5 – 15.7)	8.4 (6.2 – 10.6)	7.8 (5.2 – 10.4)	0.23
	Rice-diet	7.1 (4.4 – 9.8)	8.5 (5.5 – 11.5)	9.6 (6.5 – 12.7)	

Western confectionery	No-rice-diet	20.4 (11.1 – 29.8)	19.0 (12.0 – 26)	21.2 (11.1 – 31.3)	0.42
	Rice-diet	21.9 (15.3 – 28.4)	28.4 (16.0 – 40.7)	28.7 (18.4 – 39.0)	
Japanese confectionery	No-rice-diet	10.3 (6.9 – 13.7)	11.8 (7.5 – 16.0)	11.1 (6.4 – 15.8)	0.95
	Rice-diet	7.4 (4.0 – 10.8)	8.9 (5.0 – 12.8)	8.8 (5.2 – 12.4)	
Rice crackers	No-rice-diet	19.4 (12.1 – 26.7)	15.8 (10.9 – 20.7)	17.4 (10.4 – 24.4)	0.60
	Rice-diet	16.6 (12.0 – 21.1)	15.8 (10.4 – 21.1)	18.1 (10.5 – 25.7)	
Ice cream	No-rice-diet	22.9 (14.0 – 31.9)	15.3 (8.0 – 22.5)	13.6 (5.8 – 21.5)	0.78
	Rice-diet	20.2 (11.1 – 29.3)	12.6 (4.3 – 21.0)	14.0 (6.8 – 21.2)	
Citrus	No-rice-diet	13.3 (6.1 – 20.6)	27.3 (13.3 – 41.2)	33.2 (17.1 – 49.3)	0.60
	Rice-diet	12.7 (0.6 – 24.9)	22.1 (9.1 – 35.2)	32.7 (15.3 – 50.2)	
Persiommon/straw berry	No-rice-diet	8.3 (4.4 – 12.3)	16.6 (9.6 – 23.6)	14.5 (7.1 – 21.9)	0.088
	Rice-diet	13.8 (5.5 – 22.1)	15.2 (5.5 – 25)	20.7 (6.9 – 34.5)	
Other fruits	No-rice-diet	23.3 (13.9 – 32.6)	27.2 (17.5 – 36.9)	21.2 (14.4 – 28.0)	0.46
	Rice-diet	35.2 (20.3 – 50.2)	32.0 (14.9 – 49.2)	31.3 (16.1 – 46.6)	
Mayonnaise	No-rice-diet	7.1 (5.3 – 8.9)	7.4 (5.7 – 9.2)	6.2 (4.7 – 7.7)	0.11
	Rice-diet	7.7 (5.5 – 9.8)	5.6 (4.2 – 7.0)	6.2 (4.0 – 8.4)	
Bread	No-rice-diet	37.1 (29.6 – 44.6)	79.3 (63.5 – 95.0)	73.0 (59.2 – 86.8)	**<0.0001**
	Rice-diet	27.7 (20.0 – 35.3)	6.2$^{\#\#\#}$ (3.2 – 9.1)	8.3$^{\#\#\#}$ (4.9 – 11.7)	
Noodles	No-rice-diet	84.1 (68.0 – 100.1)	143.7 (110.8 – 176.7)	127.2 (104.4 – 150.1)	**<0.0001**
	Rice-diet	73.7 (58.7 – 88.7)	38.1$^{\#\#\#}$ (22.8 – 53.4)	45.0$^{\#\#\#}$ (25.0 – 64.9)	
Green tea	No-rice-diet	191.1 (121.0 – 261.2)	111.0 (56.7 – 165.3)	132.5 (75.2 – 189.7)	0.085
	Rice-diet	137.9 (70.6 – 205.1)	135.3 (75.8 – 194.9)	125.9 (64.9 – 187)	
Black tea/Oolong tea	No-rice-diet	52.7 (18.2 – 87.2)	47.6 (14.3 – 80.8)	47.4 (13.8 – 81.1)	0.93
	Rice-diet	35.3 (7.6 – 63.1)	24.3 (10.9 – 37.7)	23.5 (12.3 – 34.8)	
Coffee	No-rice-diet	310.3 (246.4 – 374.2)	302.3 (243.6 – 360.9)	318.8 (260.8 – 376.8)	0.31
	Rice-diet	251.5 (187.2 – 315.7)	196.3 (135.5 – 257.0)	234.2 (172.0 – 296.4)	
Coke	No-rice-diet	60.2 (19.8 – 100.5)	31.0 (13.2 – 48.9)	47.1 (8.0 – 86.1)	0.71
	Rice-diet	45.3 (27.9 – 62.7)	33.0 (18.8 – 47.2)	45.8 (23.9 – 67.8)	

Fruit Juice	No-rice-diet	34.8 (13.5 – 56.2)	21.4 (8.2 – 34.6)	23.6 (8.6 – 38.5)	0.37
	Rice-diet	44.6 (21.7 – 67.5)	30.7 (8.9 – 52.6)	21.0 (4.4 – 37.6)	
Rice	No-rice-diet	245.7 (182.2 – 309.3)	146.1 (92.4 – 199.7)	109.2 (92.1 – 126.3)	**<0.001**
	Rice-diet	282.6 (229.4 – 335.7)	375.7### (329.2 – 422.2)	347.6### (295.4 – 399.9)	
Miso	No-rice-diet	146 (90.7 – 201.2)	109.1 (71.2 – 147.1)	94.2 (77.5 – 110.9)	0.10
	Rice-diet	134.6 (97.7 – 171.4)	179.1 (142 – 216.2)	154.5 (105.4 – 203.6)	

Values are means (95% confidence interval) (n = 31 No-rice-diet group and n = 29 Rice-diet group).
Abbreviation: BDHQ, Brief–type Self–administered Diet History Questionnaire
*p–values were calculated by repeated–measures analysis of variance to investigate interaction in group–by–time. Post–hoc multiple comparisons between Rice-diet and No-rice-diet groups at each time point were conducted using Bonferroni's test (###, p<0.001).

Table S2. Changes in food intake during intervention in rice group with/without effect on sleep quality

Food item in BDHQ	Group	Duration of intervention (g/day/1000kcal)			*p*−values*
		0 weeks	4 weeks	8 weeks	Group−by−time interaction
Low fat milk	Nonresponder	7.8 (−5.5 – 21.1)	12.0 (−15.7 – 39.7)	3.7 (−6.6 – 14)	0.60
	Responder	34.6 (−20.5 – 89.6)	35.0 (−23.5 – 93.5)	5.9 (−1.7 – 13.6)	
Normal/high fat milk	Nonresponder	30.1 (−13.6 – 73.8)	38.1 (−3.3 – 79.4)	48.8 (−2.1 – 99.6)	0.32
	Responder	46.0 (−1.0 – 93.1)	38.1 (−10.0 – 86.1)	67.2 (0.9 – 133.4)	
Chicken	Nonresponder	11.3 (10.0 – 12.7)	10 (7.2 – 12.7)	14.3 (5.6 – 23.0)	0.26
	Responder	16.5 (5.3 – 27.8)	21.7 (11.2 – 32.1)	24 (5.5 – 42.6)	
Pork/beef	Nonresponder	28.9 (7.7 – 50.0)	24.8 (15.2 – 34.4)	24.2 (14.7 – 33.7)	0.40
	Responder	35.2 (13.6 – 56.8)	23.3 (13.1 – 33.6)	32 (15.7 – 48.3)	
Ham/Sausage	Nonresponder	8.4 (3.6 – 13.1)	6.2 (2.7 – 9.7)	7.2 (3.6 – 10.8)	0.54
	Responder	8.7 (−0.2 – 17.6)	10.0 (3.2 – 16.7)	10.3 (0.7 – 19.9)	
Liver	Nonresponder	1.4 (−1.3 – 4.2)	0.5 (−0.8 – 1.7)	0.4 (−0.7 – 1.5)	0.66
	Responder	0.5 (−0.7 – 1.6)	0.3 (−0.5 – 1.1)	0.0	
Squid/Octpus/ Shrimp/Shellfish	Nonresponder	14.7 (2.3 – 27.0)	9.4 (5.3 – 13.5)	10.6 (6.5 – 14.7)	0.48
	Responder	12.6 (9.8 – 15.4)	10.2 (6.4 – 14.0)	17.1 (4.3 – 29.9)	
Fish(with edible bones)	Nonresponder	2.7 (−2.5 – 7.8)	0.8 (−1.4 – 3)	2.7 (−2.5 – 7.8)	0.25
	Responder	2.8 (−0.7 – 6.4)	4.2 (0.0 – 8.4)	1.3 (−0.8 – 3.4)	
Tuna(canned)	Nonresponder	1.9 (−1.8 – 5.7)	0.6 (−1.0 – 2.2)	1.5 (−0.2 – 3.3)	1.00
	Responder	5.2 (−0.3 – 10.7)	3.9 (−2.5 – 10.3)	4.7 (−0.9 – 10.3)	
Dried fish	Nonresponder	16.4 (2.0 – 30.7)	17.4 (0.0 – 34.7)	16.9 (4.7 – 29.1)	0.34
	Responder	17.0 (−0.7 – 34.7)	35.6 (8.7 – 62.5)	20.5 (10.3 – 30.7)	
High−fat fish	Nonresponder	16.3 (3.5 – 29.0)	9.5 (4.9 – 14.2)	8.1 (3.6 – 12.6)	0.99
	Responder	23.7 (3.3 – 44.0)	16.8 (5.3 – 28.3)	14.4 (3.2 – 25.5)	
Low−fat fish	Nonresponder	15.0 (1.0 – 29.1)	18.8 (2.3 – 35.3)	13.7 (−1.5 – 28.9)	0.50
	Responder	14.5 (5.6 – 23.4)	22.4 (9.6 – 35.3)	20.0 (9.3 – 30.6)	
Egg	Nonresponder	29.0 (9.5 – 48.5)	33.1 (13.8 – 52.4)	32.2 (13.4 – 51.0)	**0.013**
	Responder	25.0 (8.6 – 41.4)	40.5 (14.5 – 66.6)	39.6 (18.1 – 61.2)	

Tofu	Nonresponder	28.5 (16.6 – 40.5)	22.8 (7.8 – 37.8)	23.4 (11.6 – 35.2)	0.91
	Responder	32.6 (7.4 – 57.7)	30.7 (−3.8 – 65.2)	27.0 (5.2 – 48.8)	
Fermented soybean	Nonresponder	10.9 (−2.4 – 24.2)	15.0 (−2.5 – 32.5)	8.0 (0.4 – 15.6)	0.79
	Responder	22.6 (8.5 – 36.7)	23.0 (6.7 – 39.3)	18.3 (7.1 – 29.4)	
Potato	Nonresponder	35.7 (14.4 – 57.0)	35.7 (14.4 – 57.0)	21.9 (3.0 – 40.9)	0.093
	Responder	42.9 (11.4 – 74.5)	81.4 (−19.4 – 182.2)	61.1 (15.4 – 106.8)	
Pickled vegetables (greens, carrots, etc.)	Nonresponder	3.8 (−0.3 – 8.0)	4.7 (−0.7 – 10.1)	4.8 (0 – 9.7)	0.66
	Responder	4.6 (−1.2 – 10.4)	6.3 (−2.2 – 14.9)	8.5 (2.6 – 14.4)	
Pickled vegetables (light−colored vegetables)	Nonresponder	5.8 (−5.5 – 17.1)	7.8 (2.1 – 13.5)	12.3 (−10.1 – 34.7)	0.94
	Responder	9.5 (−5.8 – 24.7)	14.1 (−6.6 – 34.8)	16.7 (−3.7 – 37.0)	
Fresh vegetables	Nonresponder	18.3 (3.9 – 32.7)	15.1 (13.4 – 16.9)	13.1 (7.5 – 18.7)	0.58
	Responder	25.9 (13.9 – 37.8)	40.8 (12.8 – 68.7)	29.3 (19.1 – 39.5)	
Greens	Nonresponder	14.9 (0.8 – 28.9)	19.4 (5.3 – 33.6)	13.7 (3.9 – 23.4)	0.70
	Responder	19.9 (9.3 – 30.5)	17.6 (3.2 – 32)	19.5 (0.9 – 38.2)	
Cabbage	Nonresponder	22.8 (15.3 – 30.4)	16.9 (6.2 – 27.6)	19.6 (8.6 – 30.6)	0.35
	Responder	30.6 (15.6 – 45.7)	29.4 (16.9 – 41.9)	36.9 (21.8 – 52.0)	
Carrots/pumpkins	Nonresponder	13.2 (8.1 – 18.3)	11.7 (4.7 – 18.7)	12.7 (8.5 – 17.0)	0.83
	Responder	16.2 (3.5 – 28.8)	12.2 (1.7 – 22.7)	13.8 (0.5 – 27.0)	
Radish/Turnip	Nonresponder	12.2 (1.5 – 22.8)	14.7 (4.5 – 25.0)	13.7 (6.4 – 21.1)	0.69
	Responder	18.3 (2.7 – 33.9)	18.7 (1.8 – 35.7)	24.1 (5.9 – 42.4)	
Other root vegetables	Nonresponder	24.1 (9.2 – 39.0)	25.0 (5.3 – 44.8)	24.6 (4.6 – 44.5)	0.39
	Responder	48.0 (19.9 – 76.0)	50.6 (2.1 – 99.0)	33.0 (9.9 – 56.1)	
Tomato	Nonresponder	24.4 (9.2 – 39.5)	17.8 (−1.6 – 37.2)	16.5 (4.1 – 28.9)	0.17
	Responder	24.3 (6.0 – 42.6)	19.9 (0.6 – 39.2)	26.9 (6.0 – 47.8)	
Mushrooms	Nonresponder	5.9 (0.7 – 11.0)	4.7 (0.3 – 9.1)	9.0 (−0.8 – 18.8)	0.18
	Responder	13.6 (9.0 – 18.2)	10.2 (4.5 – 15.9)	11.4 (4.9 – 18.0)	
Seaweeds	Nonresponder	3.2 (1.1 – 5.2)	5.4 (1.4 – 9.3)	3.1 (0.5 – 5.8)	0.41
	Responder	5.6 (0.2 – 11.1)	5.2 (1.0 – 9.3)	8.7 (1.0 – 16.5)	
Western confectionery	Nonresponder	22.0 (1.0 – 43.0)	18.2 (8.4 – 28.0)	25.9 (8.7 – 43.0)	0.31

	Responder	28.8 (8.7 – 48.9)	54.3 (2.2 – 106.3)	42.0 (0.4 – 83.6)	
Japanese confectionery	Nonresponder	6.8 (−1.2 – 14.8)	5.4 (−3.4 – 14.3)	5.4 (2.9 – 7.9)	0.72
	Responder	12.7 (−2.4 – 27.8)	14.2 (−0.8 – 29.1)	14.3 (0.2 – 28.5)	
Rice crackers	Nonresponder	12.3 (3.9 – 20.7)	13.0 (6.0 – 20.0)	13.0 (6.0 – 20.0)	0.12
	Responder	19.1 (4.0 – 34.2)	19.6 (1.3 – 37.9)	28.5 (−2.0 – 59.1)	
Ice cream	Nonresponder	9.6 (1.0 – 18.3)	6.4 (−2.1 – 14.8)	8.1 (−2.1 – 18.3)	0.66
	Responder	22.4 (−2.3 – 47.2)	24.6 (−12.7 – 61.9)	29.3 (3.7 – 54.9)	
Citrus	Nonresponder	5.9 (0.5 – 11.3)	14.8 (−4.4 – 34)	19.6 (−13.5 – 52.8)	0.30
	Responder	29.3 (−29.4 – 88.0)	41.9 (−15 – 98.9)	67.3 (−2.4 – 137)	
Persiommon/Strawberry	Nonresponder	3.5 (−0.5 – 7.4)	8.5 (−7.5 – 24.4)	7.2 (0.8 – 13.7)	0.87
	Responder	17.8 (−2.6 – 38.2)	25.7 (−5.8 – 57.3)	37.1 (−19.2 – 93.5)	
Other fruits	Nonresponder	18.2 (−12.1 – 48.6)	17.1 (1.2 – 33.0)	7.1 (3.4 – 10.7)	0.63
	Responder	46.0 (−12.2 – 104.2)	56.6 (−17.4 – 130.5)	52.2 (−8.6 – 112.9)	
Mayonnaise	Nonresponder	5.5 (3.2 – 7.7)	4.0 (2.7 – 5.3)	4.0 (2.3 – 5.7)	0.45
	Responder	9.7 (0.9 – 18.5)	6.4 (2.2 – 10.6)	9.8 (1.2 – 18.4)	
Bread	Nonresponder	28.2 (4.3 – 52.1)	12.4 (−2.1 – 26.9)	12.6 (−1.7 – 26.9)	0.32
	Responder	34.8 (11.7 – 58.0)	2.8 (0.3 – 5.3)	7.9 (0.5 – 15.2)	
Noodles	Nonresponder	63.8 (31.3 – 96.3)	26.2 (2.1 – 50.3)	15.6 (−0.9 – 32.2)	0.80
	Responder	77.0 (48.4 – 105.5)	26.4 (1.9 – 51.0)	21.0 (10.3 – 31.8)	
Green tea	Nonresponder	141.8 (−16.9 – 300.4)	238.2 (27.8 – 448.5)	195.0 (−0.9 – 391.0)	0.53
	Responder	135.8 (−9.1 – 280.8)	133.1 (−12.9 – 279.1)	148.6 (−61.4 – 358.6)	
Black tea/Oolong tea	Nonresponder	79.9 (−114.5 – 274.2)	6.0 (−5.3 – 17.3)	34.7 (−18.6 – 88.0)	0.19
	Responder	15.3 (−2.9 – 33.6)	9.8 (−10.6 – 30.3)	4.5 (−3.1 – 12.1)	
Coffee	Nonresponder	201.3 (7.8 – 394.9)	287.6 (12.4 – 562.8)	287.6 (12.4 – 562.8)	0.14
	Responder	354.3 (210.8 – 497.8)	283.5 (157.2 – 409.7)	283.5 (157.2 – 409.7)	
Coke	Nonresponder	35.4 (−3.9 – 74.8)	13.5 (−3.5 – 30.5)	21.7 (−13.6 – 57.1)	0.43
	Responder	42.6 (15.4 – 69.8)	38.4 (8.3 – 68.6)	57.7 (−3.3 – 118.7)	
Fruit Juice	Nonresponder	49.1 (−19.7 – 118)	13.5 (−3.5 – 30.5)	13.5 (−3.5 – 30.5)	0.41
	Responder	12.1 (−0.2 – 24.4)	24.1 (−5.9 – 54.1)	9.7 (0.1 – 19.2)	

Rice	Nonresponder	319.1 (140.7 – 497.4)	378.9 (256.8 – 501.1)	421.3 (233.5 – 609.1)	0.083
	Responder	316.6 (151.7 – 481.5)	397.4 (241.9 – 553.0)	345.8 (222.1 – 469.4)	
Miso	Nonresponder	158.8 (35.8 – 281.7)	141.5 (44.4 – 238.6)	118.5 (65.1 – 171.9)	0.19
	Responder	90.2 (59.2 – 121.1)	195.2 (94.4 – 296.1)	108.8 (60.0 – 157.6)	

Values are means (95% confidence interval) (n = 5 Nonresponder and n = 7 Responder).
Abbreviation: BDHQ, Brief−type Self−administered Diet History Questionnaire
*p−values were calculated by repeated−measures analysis of variance to investigate interaction in group−by−time. Post−hoc multiple comparisons between Responder and Nonresponder groups at each time point were conducted using Bonferroni's test.

Table S3. Identified metabolites among the test groups

ID	Compound name	Comparative Analysis			
		Post−intervention vs Pre−intervention (Responder)		Post−intervention vs Pre−intervention (Nonresponder)	
		Fold change	*p*−value	Fold change	*p*−value
C_0053	1−Methyl−4−imidazoleacetic acid	1.0	0.962	0.3	N.A.
C_0114	1−Methyladenosine	1.0	0.989	1.1	0.062
C_0076	1−Methylhistidine<br>3−Methylhistidine	0.7	0.428	0.5	0.242
C_0050	1−Methylnicotinamide	0.9	0.266	0.9	0.532
A_0049	10−Hydroxydecanoic acid	1.2	0.795	0.7	N.A.
C_0092	11−Aminoundecanoic acid	1.1	0.570	1.2	0.206
C_0090	2,6−Diaminopimelic acid	N.A.	N.A.	1<	N.A.
C_0011	2−Aminoisobutyric acid<br>2−Aminobutyric acid	1.0	0.874	0.9	0.393
A_0021	2−Hydroxy−4−methylvaleric acid	1<	N.A.	<1	N.A.
A_0006	2−Hydroxybutyric acid	0.9	0.179	0.9	0.515
A_0005	2−Hydroxyisobutyric acid	0.9	0.336	1.0	0.595
A_0014	2−Hydroxyvaleric acid	1.0	0.877	1.0	0.824
A_0028	2−Oxoglutaric acid	0.9	0.277	1.0	0.074
**A_0010**	**2−Oxoisovaleric acid**	**0.9**	**0.036**	1.0	0.588
C_0005	3−Amino−1,2,4−triazole	0.7	N.A.	N.A.	N.A.
**C_0014**	**3−Aminobutyric acid**	0.9	0.445	**1.1**	**0.002**
A_0007	3−Hydroxybutyric acid	1.2	0.671	0.9	0.898
A_0059	3−Indoxylsulfuric acid	0.9	0.447	0.9	0.370
A_0018	4−Methyl−2−oxovaleric acid<br>3−Methyl−2−oxovaleric acid	1.0	0.569	1.0	0.627
C_0038	5−Amino−4−oxovaleric acid	1<	N.A.	1<	N.A.
C_0072	5−Hydroxylysine	1.0	0.722	1.0	0.592
A_0057	5−Methoxyindoleacetic acid	1.0	0.582	1.2	0.156
**A_0017**	**5−Oxoproline**	**1.2**	**0.011**	1.1	0.068
C_0074	7−Methylguanine	1<	N.A.	1<	N.A.
A_0032	8−Hydroxyoctanoic acid<br>2−Hydroxyoctanoic acid	1.1	N.A.	<1	N.A.
C_0093	*N*,*N*-Dimethylarginine	1.0	0.944	1.0	0.674
A_0092	Adenosine 5'-diphosphate (ADP)	0.9	0.725	0.8	0.292
A_0102	ADP−ribose	N.A.	N.A.	<1	N.A.
C_0008	Alanine	1.1	0.412	1.0	0.979
C_0042	Alloisoleucine	1.0	0.948	1.2	0.154
A_0081	Adenosine 5'-monophosphate	1.0	0.833	0.9	0.354
C_0078	Arginine	0.9	0.076	0.8	0.080

C_0117	Argininosuccinic acid	0.7	N.A.	1.3	N.A.
C_0043	Asparagine	1.1	0.182	1.0	0.789
C_0046	Aspartic Acid	0.9	0.494	1.0	0.978
A_0100	Adenosine 5'-Triphosphate	0.9	0.604	0.9	0.358
C_0025	Betaine	1.0	0.789	0.9	0.439
C_0068	Betonicine	0.9	N.A.	<1	N.A.
C_0104	Butyrylcarnitine	0.8	0.159	1.2	0.090
C_0091	Caffeine	0.9	0.396	1.0	0.828
C_0071	Carnitine	1.0	0.646	1.0	0.935
A_0090	Cholic acid	0.6	N.A.	1.1	N.A.
C_0015	Choline	1.0	0.475	1.0	0.767
**A_0042**	***cis*−Aconitic acid**	**1.2**	**0.027**	1.2	0.051
A_0051	Citric acid	1.0	0.828	1.1	0.192
C_0080	Citrulline	1.0	0.838	1.0	0.601
C_0039	Creatine	0.8	0.136	0.9	0.648
C_0021	Creatinine	1.0	0.696	1.0	0.153
C_0029	Cysteine	<1	N.A.	1<	N.A.
**C_0123**	**Cysteine glutathione disulfide**	**2.6**	**0.002**	1.9	N.A.
**C_0106**	**Cystine**	**2.6**	**0.005**	1.9	0.122
C_0108	Cytidine	0.8	0.358	1.4	N.A.
A_0040	Decanoic acid	0.9	0.842	1.2	0.512
C_0017	Diethanolamine	1.3	0.489	1.3	0.508
A_0038	Dihydroxyacetone phosphate	1<	N.A.	N.A.	N.A.
C_0002	Ethanolamine	0.9	0.453	1.0	0.537
A_0025	Ethanolamine phosphate	1.0	0.964	0.8	0.461
A_0093	Guanosine 5'-diphosphate	0.7	0.167	0.6	0.174
**C_0058**	**Glutamine**	1.1	0.076	**1.1**	**0.021**
C_0060	Glutamate	0.8	0.061	0.7	0.074
A_0054	Gluconic acid	1.1	0.732	1.2	0.168
A_0068	Glucose 6−phosphate	1.1	0.619	0.8	N.A.
A_0053	Glucuronic acid Galacturonic acid	0.9	0.313	1.0	0.761
C_0119	Glutathione (GSSG)_divalent	1.5	N.A.	N.A.	N.A.
C_0003	Glycine	1.1	0.123	1.0	0.651
A_0008	Glyceric acid	1.1	0.436	0.9	0.060
C_0010	Glycerol	1.1	0.202	1.0	0.561
C_0111	Glycerophosphocholine	1.1	0.210	0.8	0.349
A_0094	Glycocholic acid	1<	N.A.	1.2	0.614
A_0101	Guanosine 5'-triphosphate	<1	N.A.	<1	N.A.
C_0079	Guanidinosuccinic acid	0.9	0.343	0.9	0.197
C_0023	Guanidoacetic acid	1.0	0.507	1.0	0.815

A_0011	Hexanoic acid	<1	N.A.	1.0	N.A.
A_0045	Hippuric acid	1.4	0.265	0.6	0.513
C_0065	Histidine	1.0	0.987	1.0	0.369
C_0088	Homoarginine	1.0	0.267	0.8	0.161
C_0089	Homocitrulline	0.9	0.664	<1	N.A.
A_0046	Homovanillic acid	1.1	0.110	1.0	0.650
C_0037	Hydroxyproline	0.9	0.278	1.0	0.912
C_0018	Hypotaurine	1.0	0.693	1.0	0.872
C_0049	Hypoxanthine	1.2	0.346	1.1	0.846
C_0041	Isoleucine	1.0	0.860	0.9	0.514
C_0066	Imidazolelactic acid	0.9	0.650	0.9	0.530
A_0082	Inosine 5′-monophosphate	1<	N.A.	0.7	N.A.
C_0067	Indole−3−acetaldehyde	1.0	0.892	1<	N.A.
A_0044	Indole−3−acetic acid	1.2	0.235	0.8	0.601
C_0113	Inosine	N.A.	N.A.	<1	N.A.
A_0016	Isethionic acid	1.0	0.503	1.0	0.742
**C_0103**	**Isobutyrylcarnitine**	0.8	0.188	**0.6**	**0.043**
**A_0050**	**Isocitric acid**	1.1	0.228	**1.3**	**$2.8x10^{-4}$**
C_0110	Isovalerylcarnitine	0.9	N.A.	N.A.	N.A.
C_0098	Kynurenine	1.0	0.631	0.9	0.291
A_0004	Lactic acid	1.7	0.161	1.7	0.294
A_0056	Lauric acid	1.0	0.125	1.0	0.727
C_0040	Leucine	1.0	0.840	0.9	0.386
C_0059	Lysine	1.0	0.568	1.0	0.625
A_0022	Malic acid	1.1	0.381	1.1	0.160
C_0061	Methionine	1.0	0.801	0.8	0.373
C_0073	Methionine sulfoxide	1.0	0.796	0.8	0.409
A_0061	Myristoleic acid	0.8	0.185	0.9	0.982
C_0013	*N*,*N*−Dimethylglycine	1.0	0.758	0.9	0.491
A_0020	*N*−Acetyl−β−alanine	0.9	0.526	0.9	0.583
A_0019	*N*−Acetylalanine	1.1	0.301	0.9	0.420
A_0043	*N*−Acetylaspartic acid	0.9	0.450	1.1	N.A.
C_0101	*N*−Acetylgalactosamine *N*−Acetylmannosamine *N*−Acetylglucosamine	1.0	0.921	1.0	0.453
A_0074	*N*−Acetylglucosamine 1−phosphate	1<	N.A.	0.6	N.A.
A_0012	*N*−Acetylglycine	1.0	0.977	1.0	0.892
C_0036	*N*−Acetylputrescine	0.8	N.A.	1.1	N.A.
C_0034	*N*−Methylproline	1.1	0.834	0.2	0.776
C_0064	*N*^{1}−Methyl−4−pyridone−5−carboxa mide	1.1	N.A.	N.A.	N.A.

A_0071	*N^{2}*−Phenylacetylglutamine	0.7	0.397	1.1	0.819
**C_0077**	***N^{5}*−Ethylglutamine**	0.9	0.504	**0.6**	**0.038**
C_0069	*N^{6}*−Methyllysine	1.0	0.980	0.9	0.074
C_0086	*N^{8}*−Acetylspermidine	0.9	0.830	1.2	N.A.
C_0030	Nicotinamide	1.2	0.351	0.9	0.562
C_0087	N_{ω}-Methylarginine	0.8	0.122	1.2	N.A.
C_0096	*O*−Acetylcarnitine	1.1	0.523	1.0	0.713
C_0070	*O*−Acetylhomoserine<br>2−Aminoadipic acid	0.9	0.275	1.0	0.890
A_0024	*o*−Hydroxybenzoic acid	1<	N.A.	N.A.	N.A.
A_0026	Octanoic acid	1.0	N.A.	1.5	N.A.
C_0116	Octanoylcarnitine	1.0	0.445	1.3	0.214
**C_0044**	**Ornithine**	**1.3**	**0.022**	1.3	0.052
A_0030	*p*−Anisic acid<br>*o*−Hydroxyphenylacetic acid<br>Mandelic acid<br>Phenoxyacetic acid	0.9	N.A.	1.2	N.A.
A_0060	Pantothenic acid	0.9	0.077	1<	N.A.
C_0082	Paraxanthine	0.9	0.274	1.1	0.872
A_0031	Pelargonic acid	1.1	0.163	1.0	0.904
A_0034	Perillic acid	1.2	0.224	1.1	0.551
C_0075	Phenylalanine	1.0	0.833	1.0	0.576
A_0058	Phosphocreatine	0.9	0.511	0.9	0.260
C_0085	Phosphorylcholine	1.1	0.421	0.8	0.379
C_0035	Pipecolic acid	0.8	0.511	1.0	0.972
C_0022	Proline	1.0	0.679	0.9	0.402
A_0003	Pyruvic acid	1.4	0.055	1.5	0.070
A_0052	Quinic acid	0.9	0.914	1.0	0.914
A_0063	Ribose 5−phosphate	1.1	N.A.	1<	N.A.
A_0064	Ribulose 5−phosphate	N.A.	N.A.	1<	N.A.
**C_0047**	***S*−Methylcysteine**	**1.8**	**0.013**	1.5	0.102
C_0007	Sarcosine	0.9	0.489	0.9	0.352
C_0094	*N,N'*-dimethylarginine	1.0	0.649	1.0	0.967
C_0016	Serine	1.1	0.283	1.0	0.969
C_0105	L-Seryl-L-glutamic acid	0.8	0.177	0.9	0.606
C_0081	Serotonin	1.2	0.507	0.9	0.970
C_0055	Stachydrine	1.5	0.416	0.3	0.313
A_0013	Succinic acid	0.9	0.850	1.1	0.609
**A_0070**	**Sulfotyrosine**	1.0	0.739	**1.2**	**0.004**
C_0031	Taurine	1.0	0.726	1.1	0.725
A_0033	Terephthalic acid	1.0	0.532	0.9	0.246
C_0083	Theobromine	0.8	0.664	1.2	0.689

C_0045	Thiaproline	1.1	0.536	0.8	0.448
C_0026	Threonine	1.0	0.927	0.9	0.248
A_0023	Threonic acid	0.9	0.219	0.9	0.101
C_0062	Triethanolamine	1.1	0.463	1.1	0.473
C_0004	Trimethylamine *N*−oxide	0.8	0.368	0.5	0.386
C_0097	Tryptophan	1.0	0.811	1.0	0.845
C_0084	Tyrosine	1.0	0.711	1.0	0.615
C_0001	Urea	0.9	0.222	0.9	0.509
**A_0036**	**Uric acid**	**0.9**	**0.022**	1.0	0.450
C_0109	Uridine	0.9	0.346	1.0	0.765
A_0098	Uridine-5'-triphosphate	0.8	0.194	0.9	0.633
C_0024	Valine	0.9	0.243	1.0	0.613
A_0015	XA0003	N.A.	N.A.	1<	N.A.
A_0027	XA0004	1.0	N.A.	0.8	0.099
A_0041	XA0013	0.6	0.207	0.9	0.868
**A_0062**	**XA0027**	**0.8**	**0.034**	1.1	0.816
A_0065	XA0033	0.8	0.505	0.9	0.864
A_0066	XA0036<br>Ascorbate 2−sulfate	0.9	0.106	1.0	0.672
A_0029	Xanthine	1<	N.A.	<1	N.A.
C_0033	XC0016	0.9	0.295	1.0	0.984
C_0054	XC0029	0.8	0.302	0.8	0.326
C_0099	XC0061	1.0	0.325	0.7	0.133
C_0100	XC0065	1.2	N.A.	0.8	N.A.
**C_0118**	**XC0120**	**2.9**	**8.1×10^{-4}**	**2.4**	**0.036**
C_0121	XC0132	0.9	0.379	1.0	0.156
C_0009	β−Alanine	1.0	0.701	1.0	0.541
C_0056	γ−Butyrobetaine	1.0	0.411	1.0	0.548

Comparison between pre− and post−intervention in the Responder and the Nonresponder was performed by Welch's t−tests (n = 5 Nonresponder and n = 7 Responder).
